# Pulsed Field Ablation Terminated Atrial Fibrillation Triggered by Persistent Left Superior Vena Cava: A Case Report

**DOI:** 10.1002/ccr3.72152

**Published:** 2026-02-28

**Authors:** Yun Xie, Changjian Lin, Liqun Wu

**Affiliations:** ^1^ Department of Cardiovascular Medicine, Ruijin Hospital Shanghai Jiaotong University School of Medicine Shanghai China

**Keywords:** atrial fibrillation, case report, persistent left superior vena cava, pulsed field ablation

## Abstract

A 73‐year‐old patient with paroxysmal atrial fibrillation (AF) and a history of two ablation procedures was referred to our center due to recurrence. Persistent left superior vena cava (PLSVC) was revealed in preprocedural echocardiography and computed tomography. A circular array Pulsed Field Ablation (PFA) catheter was applied for the re‐intervention. Incessant AF was induced during the procedure. PFA successfully terminated AF within the distal part of PLSVC, where high‐frequency fragmented electrograms were found, demonstrating the efficacy of circular array PFA catheter for the ablation within PLSVC.

## Key Clinical Message

As a relatively common vascular anomaly, PLSVC may serve as an important non‐PV trigger focus, warranting careful evaluation, especially during re‐intervention procedures. PFA may offer a more effective and safer alternative to traditional thermal ablation for lesions within the PLSVC, thereby broadening the clinical application of PFA catheters beyond PVI.

## 1 Introduction

Persistent left superior vena cava (PLSVC) is the most common congenital anomaly of the thoracic venous system, with an incidence of approximately 0.5% in the general population [1]. Its embryological remnant, ligament of Marshall (LOM), is a well‐recognized non‐pulmonary vein (PV) trigger of atrial fibrillation (AF) [2]. When the normal regression to the LOM fails, the resulting PLSVC has been reported to exhibit arrhythmogenic potential in both paroxysmal and persistent AF [3]. Traditionally, radiofrequency (RF) energy has been employed for ablation within the PLSVC, targeting either elimination of AF triggers or complete electrical isolation of the vessel [4]. Considering the difficulty in achieving either target, as well as the significant safety concerns of RF ablation within coronary sinus, such cases are often technically challenging.

Pulsed field ablation (PFA) is a novel nonthermal ablation technology that induces cardiomyocyte death by creating irreversible electroporation through controlled electrical field [5]. Compared with RF energy, PFA offers high myocardial selectivity, which results in a superior safety profile in previous studies without compromising efficacy [6]. However, the use of PFA for non‐PV foci has not yet been systematically investigated.

Here, we report a case of successful AF termination achieved during PLSVC ablation using a circular array PFA catheter.

## 2 Case History

A 73‐year‐old female patient was referred to our center with frequent episodes of palpitations. Electrocardiogram confirmed a diagnosis of paroxysmal AF. She had previously undergone two catheter ablation procedures for AF, the first, a cryoballoon ablation performed 5 years earlier, and the second an RF ablation conducted 2 years prior. Both procedures were performed at other institutions, with procedure details unavailable. Despite these interventions, the patient continued to experience recurrent tachyarrhythmia episodes, which worsened over the preceding 6 months. As the symptoms were not relieved by amiodarone treatment, a re‐intervention using the novel energy source was planned.

## 3 Investigations and Treatment

**FIGURE 1:**
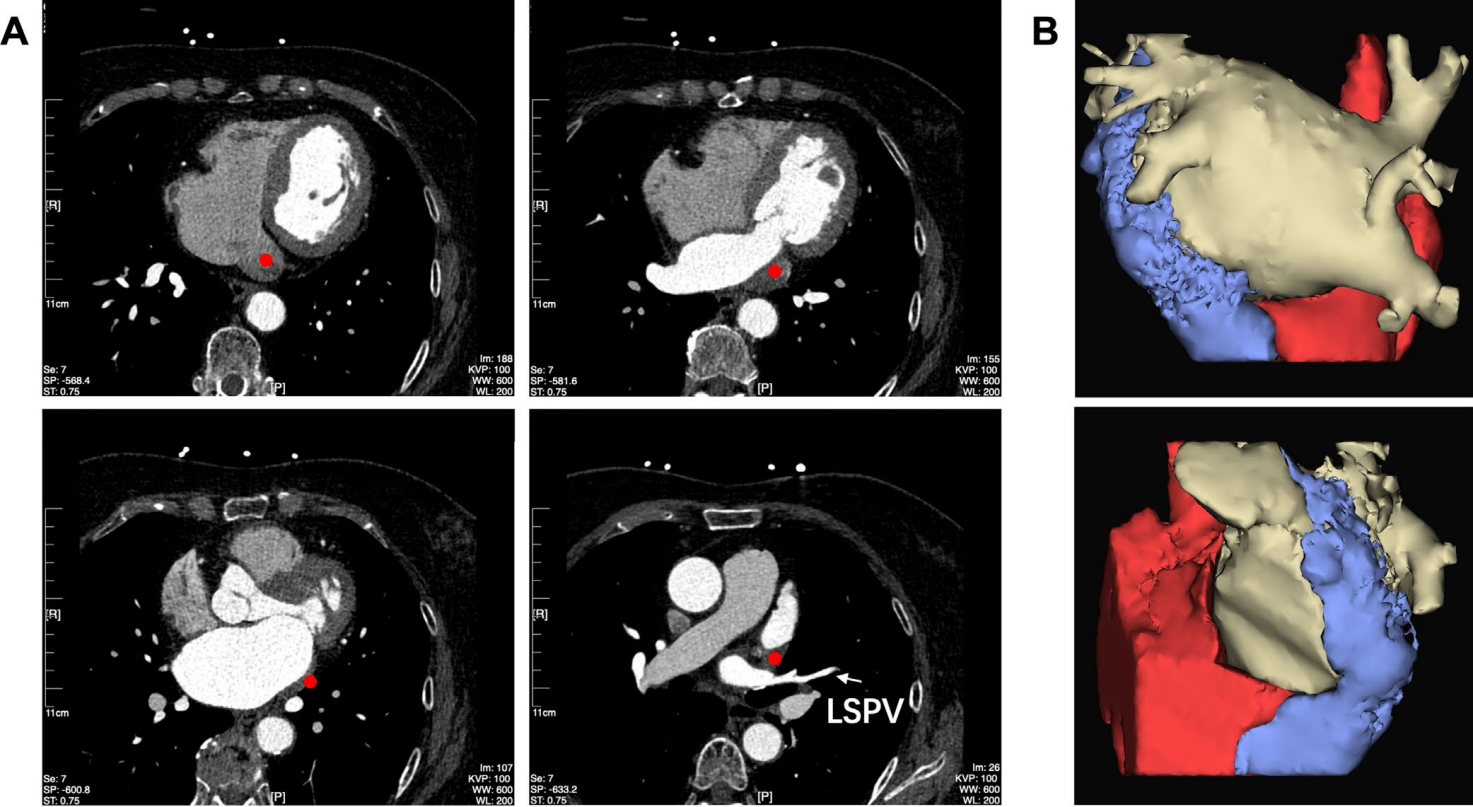
Preprocedural imaging confirmed the presence of PLSVC. (A) Coronary computed tomography angiography (CCTA) slice images, with red dots on each slice indicating the course of the PLSVC. (B) Three‐dimensional CCTA reconstruction showing the left atrium (white), right atrium and coronary sinus ostium (red), and PLSVC (blue).

Transthoracic echocardiography revealed mild left atrium (LA) enlargement and raised suspicion of a PLSVC. Coronary computed tomography angiography was subsequently performed, enabling three‐dimensional reconstruction that demonstrated a markedly dilated coronary sinus (CS) ostium and confirmed the presence of PLSVC (Figure 1).

During the procedure, the patient was placed under general anesthesia. A circular array PFA catheter (PulseSelect, Medtronic, MN) was introduced into the LA via a 10F steerable sheath (FlexCath, Medtronic, MN) under fluoroscopic guidance. Simultaneous three‐dimensional electroanatomic mapping (EAM) was performed using Ensite Precision system (St Jude Medical Inc., St. Paul, MN). The patient was initially in sinus rhythm, and delayed electrical potentials were noted in the left superior pulmonary vein (LSPV). The other pulmonary veins were found to be electrically isolated.

PFA of LSPV was performed on both the ostial and antral level. While wide antral electrical isolation was achieved in all the PVs, atrial burst pacing induced AF without cessation. AF was reinitiated immediately following electrical cardioversion. The PFA catheter was then withdrawn to the right atrium. Electrical isolation of superior vena cava (SVC) was performed to rule out the right‐side SVC as the potential AF trigger, which had no observable effect.

**FIGURE 2:**
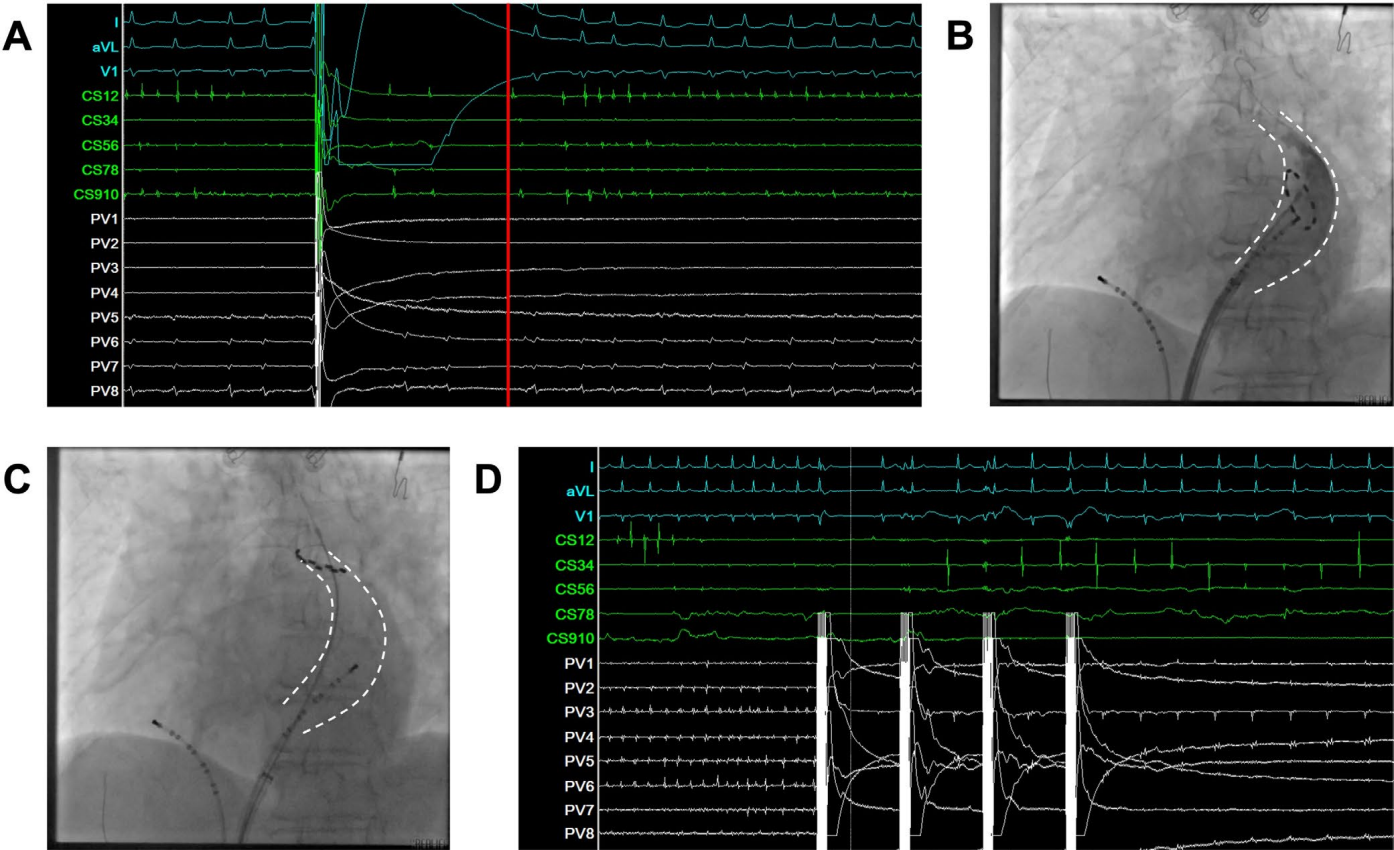
PFA ablation within the PLSVC. (A) Intracardiac electrogram demonstrating the earliest activation in the distal coronary sinus following electrical cardioversion. (B) Selective angiography of the PLSVC in the left anterior oblique (LAO) view. (C) Fluoroscopic LAO image showing the ablation catheter positioned in the distal PLSVC, where PFA successfully terminated the arrhythmia. (D) Intracardiac electrogram illustrating immediate AF termination following PFA application within the PLSVC.

Another electrical cardioversion was attempted. While AF still rapidly recurred, the initial two beats appeared to originate from the distal CS (Figure 2A), suggesting that the PLSVC was the likely source of the arrhythmia. The PFA catheter was subsequently positioned within the CS, and angiography delineated the course of the PLSVC (Figure 2B). Rapid electrical activity was detected within the CS, we thus attempted a single PFA application at the proximal and mid portion of the PLSVC, which failed to terminate AF. The catheter was then carefully advanced into the distal PLSVC over a guidewire. At the level corresponding to the LSPV, rapid electrical activity was presented (Figure 2C). A single PFA application at this site resulted in immediate termination of tachycardia and restoration of sinus rhythm (Figure 2D). The catheter was subsequently rotated incrementally by 3–5 electrodes to perform consolidation ablation. Similar manipulations were delivered to adjacent areas, totaling 12 PFA applications.

## 4 Outcome and Follow‐up

AF was uninducible following ablation within PLSVC. No diaphragmatic movement dysfunction was observed under fluoroscopy, and no ST‐segment changes were noted on the 12‐lead electrocardiogram during or after the procedure. The patient was discharged next day, and remained in stable sinus rhythm without antiarrhythmic treatment during the 3‐month follow‐up period.

## 5 Discussion

As a novel ablation modality, PFA catheters are increasingly being utilized for the ablation of extra‐PV lesions [7, 8]. However, PFA within the PLSVC has only been described in a few isolated cases [9, 10, 11]. FARAPULSE (Boston Scientific, Menlo Park, CA) catheter was employed most frequently, while PulseSelect catheter was also applied in some cases. Our case further demonstrated the feasibility of PLSVC ablation using a fixed‐loop PFA catheter [12]. Importantly, this represents the first documented case of real‐time AF termination during PLSVC ablation with PFA, providing direct electrophysiological evidence of the arrhythmia source and confirming the efficacy of this approach.

The arrhythmogenic potential of PLSVC has been fully recognized. The first five cases of PLSVC‐associated AF were reported by Hsu et al. [13], who proposed that the continuing presence of pacemaker tissue and hence ectopic pacemaker activity, attributed to its embryological origin from the common cardinal vein, was the major mechanism. Minami et al. [14] further investigated the localization of triggers within PLSVC, identifying that 81.3% originated from its distal segment of the vessel in 14 patients. Consistent with these findings, our case also demonstrated effective ablation exclusively at the distal PLSVC, suggesting that future procedural strategies could be optimized accordingly in AF cases with PLSVC. Both the PLSVC and the CS share multiple electrical connections with the LA endocardium. Traditionally, complete electrical isolation of the PLSVC requires multiple RF energy deliveries, which is time‐consuming and associated with considerable safety concerns. Current commercially‐available PFA catheters are predominantly designed in a large‐interface, single‐shot pattern, which offer superior efficiency when treating broad anatomical targets [15], making them a potentially favorable option when electrical isolation of PLSVC is required.

Safety remains a key concern when treating PLSVC‐mediated AF with conventional thermal energy. Reported complications include CS perforation and stenosis [3]. Cryoballoon ablation has also been attempted [16], achieving faster electrical isolation but increasing the risk of left‐sided phrenic nerve palsy and limiting precise targeting of focal triggers within PLSVC. Owing to its high myocardial selectivity, PFA minimizes collateral injury to adjacent structures compared with RF or cryoablation. In clinical practice, we favor ablation limited to the focal trigger site within PLSVC, rather than complete electrical isolation, to maximally preserve normal tissue function. As demonstrated in this case. However, optimal parameters for tissue contact and the appropriate ablation dosage during PFA within PLSVC remained to be defined. Further clinical exploration is needed to confirm the achievement of durable transmural lesions and to refine procedural protocols. This uncertainty was the rational for performing multiple consolidation ablations in this case. Additionally, coronary artery spasm has been identified as a potential complication when PFA is applied in this region [17]. In certain cases, prophylactic intracoronary administration of organic nitrates has been performed before ablation to mitigate this risk [10]. Although the incidence and potential severity of vasospasm remain unclear, continuous hemodynamic and electrocardiographic monitoring during the procedure is essential for safety.

In conclusion, this case illustrates successful ablation of AF originating from PLSVC using a circular array PFA catheter, with real‐time AF termination observed during energy delivery. As a relatively common vascular anomaly, PLSVC may serve as an important non‐PV trigger focus, warranting careful evaluation, especially during re‐intervention procedures. Our case highlights the potential advantages of PFA in managing PLSVC‐related AF and support its expanding role beyond pulmonary vein isolation.

## Author Contributions

**Yun Xie:** investigation, writing – original draft. **Changjian Lin:** data curation, investigation. **Liqun Wu:** conceptualization, supervision, validation.

## Funding

The study is supported by the foundation of Specialized Disease Diagnosis and Treatment Centers of Shanghai Jiao Tong University School of Medicine (YW0019).

## Consent

The authors confirm that written informed consent for the submission and publication of this case report, including all accompanying images and associated text, was obtained from the patient in accordance with the COPE guidelines.

## Conflicts of Interest

The authors declare no conflicts of interest.

## Data Availability

The data underlying this article will be shared on reasonable request to the corresponding author.
